# The role of germline and somatic mutations in predicting cancer-associated thrombosis: a narrative review

**DOI:** 10.1097/MOH.0000000000000861

**Published:** 2025-01-28

**Authors:** Vincent Lanting, Merel Oskam, Hanneke Wilmink, Pieter W. Kamphuisen, Nick van Es

**Affiliations:** a Amsterdam UMC, University of Amsterdam, department of Vascular Medicine, Amsterdam Cardiovascular Sciences, Amsterdam; b Tergooi MC, department of Internal Medicine, Hilversum; c Amsterdam UMC, University of Amsterdam, department of Oncology, Cancer Center Amsterdam, Amsterdam, The Netherlands

**Keywords:** cancer-associated thrombosis, genetics, germline, prediction, somatic

## Abstract

**Purpose of review:**

Patients with cancer have an increased risk of venous thromboembolism (VTE). Guidelines suggest to use risk assessment tools to guide decisions about thromboprophylaxis, but current tools have modest discriminatory ability. Genetic information from the germline or tumor has the potential to improve VTE prediction. Here, we provide a clinical overview of the current role of genetics in cancer-associated VTE.

**Recent findings:**

Germline mutations, such as factor V Leiden and prothrombin G20210A, are associated with a 2- to 2.5-fold increased VTE risk in patients with cancer. Tumor-specific somatic mutations also contribute to VTE risk, such as *ALK* rearrangements increasing the risk in nonsmall cell lung cancer and *IDH1* mutations decreasing the risk in gliomas. Other somatic mutations associated with VTE independent of tumor type include *KRAS*, *STK11*, *MET*, *KEAP1*, *CTNNB1*, and *CDKN2B*. Incorporating data on germline or somatic mutations in risk scores improves discriminatory ability compared with the Khorana score.

**Summary:**

Specific germline and somatic mutations are associated with an increased VTE risk in patients with cancer and potentially improve performance of clinical risk scores. The increasing and widespread use of genetic testing in cancer care provides an opportunity for further development of prediction models incorporating genetic predictors.

## INTRODUCTION

The risk of venous thromboembolism (VTE) in patients with cancer is up to ninefold higher compared to people without cancer and is associated with increased mortality and morbidity [1–4]. While multiple mechanisms contribute to VTE in patients with cancer, the exact pathophysiology remains incompletely understood [5]. Clinically, both patient-specific factors [e.g., age, body mass index (BMI), and history of VTE] and cancer-specific factors (e.g., cancer type, stage, and treatment) are associated with an increased VTE risk [2].

Guidelines suggest to treat patients at high risk of VTE with thromboprophylaxis, if the bleeding risk is low [5–7]. The most used and widely validated risk score is the Khorana score, though it has shown modest discriminatory ability and inconsistent performance across different studies and cancer types [8–10]. A meta-analysis demonstrated that the number needed to treat of thromboprophylaxis is 25 among patients with a Khorana score of ≥2, which is still considered too high by some oncologists and hematologists [6,11]. Incorporating additional biomarkers into existing prediction models could potentially enhance their discriminatory ability.

Germline mutations in genes encoding coagulation-related proteins that lead to an increased VTE risk are referred to as inherited thrombophilia. Routine testing in patients at high risk of VTE in the general population is not recommended, unless it has important treatment consequences [12]. In the oncology population, these mutations may be useful in identifying patients with cancer at higher risk of VTE.

Somatic mutations, which occur in all dividing cells, can accumulate over time and contribute to cancer development and aging [13]. Given that cancer type is a strong predictor of VTE, tumor-specific somatic mutations may also play an important role in driving the increased risk of VTE in patients with cancer [1]. Both germline and somatic mutations could be valuable predictors for improvement of the current prediction models and improve personal VTE risk estimations.

This review provides an overview of germline and somatic mutations associated with VTE in patients with cancer and discusses the potential role of genetic predictors in current risk assessment tools. 

**Box 1:**
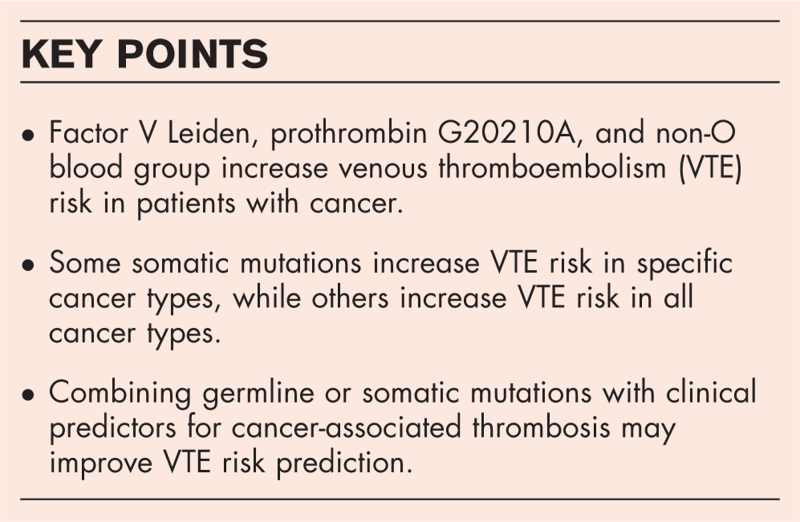
no caption available

## METHODS

MEDLINE was systematically searched up to September 2024 using terms related to genetics, cancer, and venous thromboembolism. Detailed search terms and selection criteria are provided in Table 1, Supplemental Digital Content.

## SINGLE GERMLINE MUTATIONS

The factor V Leiden mutation leads to protein C resistance, resulting in reduced factor V inhibition. This mutation is prevalent in approximately 5% of the white population, but is rare in other ethnic populations [14]. The risk of developing VTE is a 4-5-fold higher in patients with heterozygosity for factor V Leiden compared to the general population [15,16]. A meta-analysis of 18 studies with a total of 8408 patients with cancer demonstrated that factor V Leiden was associated with a more than twofold increased risk of VTE [odds ratio (OR): 2.28, 95% confidence interval (CI): 1.51–3.48] (Table 1) [17^▪^]. Although this relative increase in risk in patients with cancer appears to be significantly lower than in the general population, the absolute increase in risk due to factor V Leiden may still be clinically significant in patients with cancer given their much higher baseline risk of VTE [2,15].

**Table 1:** Germline mutations and their associations with VTE

Mutation	Study	Study design	Number of patients	Cancer type	Risk
Factor V Leiden	Roy *et al.*[17^▪^]	Meta-analysis	8408	Various	OR 2.28 (CI 95%:1.51–3.48)
Factor V Leiden	Guman *et al.*[34]	Retrospective cohort study	36 150	Various	Adjusted sHR 2.21 (CI 95%:1.77–2.76)
Prothrombin G20212A	Roy *et al.*[17^▪^]	Meta-analysis	2974	Various	OR 2.14 (95% CI:1.14–4.03)
Prothrombin G20212A	Guman *et al.*[34]	Retrospective cohort study	36 150	Various	Adjuted sHR 1.25 (95% CI:0.84–1.87)
ABO blood group	Roy *et al.*[17^▪^]	Meta-analysis	4719	Various	OR 1.56 (95% CI: 1.28–1.90)
ABO blood group	Guman *et al.*[34]	Retrospective cohort study	36 150	Various	Adjusted sHR 1.28 (95% CI: 1.13–1.45)
MTHFR C677T	Roy *et al.*[17^▪^]	Meta-analysis	741	Various	OR 1.50 (95% CI:1.01–2.24)
PAI-1 4G4G	Roy *et al.*[17^▪^]	Meta-analysis	480	Various	OR 1.46 (95% CI:0.65–3.29)
PAI-1 4G4G	Wang *et al.*^a^[59]	Prospective cohort study	214	Lung cancer	OR 2.62 (95% CI:1.19–5.75)
PAI-1 4G5G	Roy *et al.*[17^▪^]	Meta-analysis	697	Various	OR 0.93 (95% CI:0.61–1.40)

a Included in the meta-analysis of Roy *et al.* CI, confidence interval; HWE, Hardy–Weinberg equilibrium; OR, odds ratio; sHR, subdistributional hazard ratio; VTE, venous thromboembolism.

The prothrombin G20210A mutation is a gain-of-function mutation associated with increased plasma concentrations of prothrombin [18,19]. The prevalence of this mutation in the general population is 2% and more common in white populations than in those of African American descent [20]. Patients with a prothrombin G20210A mutation have a 3-fold increased risk of VTE compared with the general population [19,21,22]. A meta-analysis of 13 studies with a total of 2974 patients with cancer showed that the prothrombin G20210A mutation was associated with a twofold increased risk of VTE (OR 2.14, 95% CI: 1.14–4.03) (Table 1) [17^▪^], similar to factor V Leiden. Therefore, the presence of this mutation may identify high-risk VTE patients, although its low prevalence limits its use in clinical practice.

Other well known inherited thrombophilias are deficiencies of antithrombin, protein C, and protein S [21,23]. However, the prevalence of these mutations in the general population is low (<1.5%) and the correlation with activity measurements can be poor, hampering translation to clinical practice. Data on the association between these mutations and VTE in patients with cancer is very limited.

Patients with non-O blood group have an increased risk of VTE, which might be caused by higher levels of Von Willebrand factor and factor VIII compared with the O blood group [24]. A meta-analysis including seven studies with 4719 patients with cancer found that patients with non-O blood had a 1.5-fold increased VTE risk (OR 1.56, 95% CI: 1.28–1.90) [17^▪^]. Given the high prevalence of non-O blood group, around 50%, it may be an important and easily measured genetic risk factor [25].

The MTHFR gene can lead to mild hyperhomocysteinemia, but is not considered a relevant risk factor for VTE, since normalizing homocysteine levels with vitamins does not lower the risk of VTE [26–28]. Testing for this mutation is therefore discouraged. Also in patients with cancer, a meta-analysis of 5 studies was unable to demonstrate a significant association between MTHFR C677T mutation and VTE (OR 1.50, 95% CI: 1.00–2.24; Table 1) [17^▪^].

Mutations in the PAI-1 gene can lead to overexpression of plasminogen activator inhibitor, which inhibits fibrinolysis and therefore may increase VTE risk. However, a meta-analysis including 4 studies showed no increased VTE risk in patients with homozygous 4G/4G PAI-1 mutations in patients with cancer (OR 1.46, 95% CI: 0.65–3.29; Table 1), strongly arguing against an important role for PAI-1 mutation as a germline predictor in this population [17^▪^].

## POLYGENIC RISK SCORES

Various single nucleotide polymorphisms (SNPs) are weak risk factors for VTE [29–33]. While these individual SNPs have limited predictive value, combining them into polygenic risk scores may help identify high-risk patients. Several polygenic risk scores have been developed for the general population, which often include well known thrombophilias, such as factor V Leiden, prothrombin gene mutation, and ABO blood group, together with many SNPs [30–33]. Guman *et al.* evaluated such scores in 36 150 patients with cancer, finding *c*-statistics ranging from 0.56 (95% CI: 0.54–0.58) for the 5-SNP score to 0.60 (95% CI: 0.58–0.62) for the 297-SNP score [34]. Combining the 297-SNP score with the cancer type classification according to the Khorana score somewhat improved the c-statistic (0.65, 95% CI: 0.63–0.67), indicating that combining genetic scores with clinical variables may improve the performance of current clinical risk assessment tools [34].

ONCOTHROMB is a clinical-genetic risk score that was developed in a cohort of 364 patients with cancer of whom 18% were diagnosed with VTE. It includes 9 SNPs, BMI, cancer site, and cancer stage. The score had good discriminatory performance in the derivation (*c*-statistic, 0.78; 95% CI: 0.74–0.782) and modest in the external validation cohort (*c*-statistic, 0.69; 95% CI: 0.63–0.74) [35], but still outperformed the Khorana score. Although these findings can be seen as promising, the score cannot be externally validated in other cohorts because details of the model were not reported [36]. Future studies combining clinical predictors with SNPs should further define the added value of germline mutations in predicting VTE risk in patients with cancer.

## SOMATIC MUTATIONS

Certain somatic mutations are frequently observed in specific tumor types, while others are prevalent across various cancers. Here, we will discuss the most studied tumor types in relation with VTE. Table 2 provides an overview of somatic mutations associated with VTE in patients with cancer.

**Table 2:** Somatic mutations and their association with VTE

Mutation	Study	Study design	Population	Cancer type	Risk	Adjusted for
KRAS	Dunbar *et al.*[39]	Retrospective cohort study	11 695	Various	HR 1.34 (95% CI: 1.09–1.64)	Age, previous VTE episode, Anticoagulation, metastatic disease, cytotoxic chemotherapy
KRAS	Jee *et al.*[56^▪▪^]	Retrospective cohort study	4141	Various	HR 1.65 (95% CI: 1.30–2.09)	Cancer type
KRAS	Ades *et al.*[47]	Retrospective cohort study	172	Metastatic Colorectal	OR 2.21 (95% CI: 1.08–4.53)	Khorana score, Treatment (Bevacizumab)
KRAS	Emilescu *et al.*[49]	Retrospective cohort study	130	Colorectal	HR 2.54 (95% CI:1.32–4.89)	Age, Sex, ECOG, Stage, Khorana, Treatment (Bevacizumab)
EGFR	Jee *et al.*[56^▪▪^]	Retrospective cohort study	4141	Various	HR 1.62 (95% CI: 1.24–2.10)	Cancer type
EGFR	Abufarhaneh *et al.*[55]	Systematic review and meta-analysis	10 564	NSCLC	RR 0.97 (95% CI: 0.69–1.34)	Not applicable
EGFR	Kapteijn *et al.*[43]	Retrospective cohort study	324	Glioblastoma	HR 1.27 (95% CI 0.56–2.87)	Not adjusted
EGFR	Huang *et al.*[44]	Prospective cohort study	131	Glioblastoma	HR 2.19 (95% CI: 1.15–4.19)	Age, Sex, ECOG score, BMI, Hemiparesis, Platelet count, D-dimer, IDH1, PTEN, P53, TERTp, MGMT promotor methylation status, EGFR amplification status
PTEN	Kapteijn *et al.*[43]	Retrospective cohort study	324	Glioblastoma	HR 1.10 (95% CI: 0.50–2.40)	Not adjusted
PTEN	Huang *et al.*[44]	Prospective cohort study	131	Glioblastoma	HR 1.81 (95% CI: 0.91–3.59)	Age, Sex, ECOG score, BMI, Hemiparesis, Platelet count, D-dimer, IDH1, PTEN, P53, TERTp, MGMT promotor methylation status, EGFR amplification status
IDH-1	Dunbar *et al.*[39]	Retrospective cohort study	487	Glioma	HR 0.26 (95% CI:0.08–0.79)	Cancer type
IDH-1	Mir Seyed Nazari *et al.*[42]	Prospective cohort study	213	Glioma	HR 0.11 (95% CI: 0.01–0.83)	Age, glioblastoma
IDH-1	Unruh *et al.*[40]	Retrospective cohort study	317	Glioma	OR 0.05 (95% CI: 0.003–0.91)	Karnofsky performance score, glioblastoma, length of hospital stay
IDH-1	Watanabe *et al.*[41]	Retrospective cohort study	165	Glioma	OR 0.10 (95% CI: 0.01–0.98)	Karnofsky performance score, podoplanin expression, age
ALK	Abufarhaneh *et al.*[55]	Systematic review and meta-analysis	10 564	NSCLC	RR 1.97 (95% CI:1.54–2.52)	Not applicable
ROS1	Xiong *et al.*[54]	Retrospective cohort study	1187	NSCLC	OR 1.29 (95% CI: 0.92–2.01)	ALK, EGFR, BRAF, V600E, PDL-1
CDKN2A	Jee *et al.*[56^▪▪^]	Retrospective cohort study	4141	Various	HR 1.84 (95% CI: 1.29–2.63)	Cancer type
CDKN2B	Dunbar *et al.*[39]	Retrospective cohort study	11 695	Various	HR 1.45 (95% CI: 1.13–1.85)	Age, previous VTE episode, Anticoagulation, metastatic disease, cytotoxic chemotherapy
STK11	Dunbar *et al.*[39]	Retrospective cohort study	11 695	Various	HR 2.12 (95% CI: 1.55–2.89)	Age, previous VTE episode, Anticoagulation, metastatic disease, cytotoxic chemotherapy
STK11	Jee *et al.*[56^▪▪^]	Retrospective cohort study	4141	Various	HR 1.61 (95% CI: 1.06–2.46)	Cancer type
MET	Dunbar *et al.*[39]	Retrospective cohort study	11 695	Various	HR 1.83 (95% CI: 1.15–2.92)	Age, previous VTE episode, Anticoagulation, metastatic disease, cytotoxic chemotherapy
KEAP1	Dunbar *et al.*[39]	Retrospective cohort study	11 695	Various	HR 1.84 (95% CI: 1.21–2.79)	Age, previous VTE episode, Anticoagulation, metastatic disease, cytotoxic chemotherapy
KEAP1	Jee *et al.*[56^▪▪^]	Retrospective cohort study	4141	Various	HR 2.50 (95% CI: 1.62–3.85)	Cancer type
CTNNB1	Dunbar *et al.*[39]	Retrospective cohort study	11 695	Various	HR 1.73 (95% CI: 1.15–2.60)	Age, previous VTE episode, Anticoagulation, metastatic disease, cytotoxic chemotherapy
CTNNB1	Jee *et al.*[56^▪▪^]	Retrospective cohort study	4141	Various	HR 2.70 (95% CI: 1.74–4.18)	Cancer type
TP53	Jee *et al.*[56^▪▪^]	Retrospective cohort study	4141	Various	HR 1.85 (95% CI: 1.53–2.23)	Cancer type
ERBB2	Jee *et al.*[56^▪▪^]	Retrospective cohort study	4141	Various	HR 1.72 (95% CI: 1.10–2.68)	Cancer type

CI, confidence interval; HR, hazard ratio; OR, odds ratio; VTE, venous thromboembolism.

### Glioblastoma

Patients with glioma, particularly glioblastoma, have a high VTE incidence (10–30%) [37]. This observation is intriguing, because glioblastoma is confined to the brain while driving remote thrombus formation. Glioma-related mutations have been linked to this increased VTE risk – the incidence is strikingly higher in patients with isocitrate dehydrogenase 1 (IDH1) wildtype tumors, which distinguishes glioblastomas from other gliomas [38]. For example, among 487 high-grade glioma patients, IDH1 mutation was associated with a 74% reduced VTE risk compared to wild-type (adjusted HR 0.26, 95% CI: 0.08–0.79) [39]. This observation was confirmed in three other studies [40–42]. The proposed mechanism of increased VTE risk is increased podoplanin expression on tumor cells in IDH1 wildtype tumors, leading to platelet activation [37]. Unruh *et al.* found that IDH1 mutations are also associated with decreased tissue factor (TF) expression [40].

EGFR and PTEN mutations are two other frequently occurring mutations that lead to increased TF expression [37]. However, results about their association with VTE in glioblastoma are conflicting. The presence of an EGFR mutation significantly increased VTE risk in a prospective cohort study of 131 glioblastoma patients (adjusted HR 2.19, 95% CI: 1.15–4.19) but not in a larger retrospective cohort study of 324 glioblastoma patients (HR 1.27, 95% CI: 0.56–2.87) [43,44]. Two studies did not find a significant association between PTEN and VTE [43,44]. Taken together, IDH1 appears to be the strongest predictor for VTE in glioma patients.

### Colorectal cancer

Colorectal cancer is considered to be a cancer type with an intermediate VTE risk. *KRAS is* a common mutation in colorectal (prevalence ∼40%), but also in pancreatic (prevalence ∼70%) and lung cancers (prevalence ∼20%) [45]. This mutation is linked to upregulation of TF on tumor cells, potentially increasing the VTE risk by shedding of tumor-derived extracellular vesicles exposing TF [46–48]. A retrospective cohort study including 172 patients with metastatic colorectal cancer found a 2-fold increased VTE risk in those with KRAS mutations (OR 2.21, 95% CI: 1.08–4.53) [47]. This association was confirmed in a small retrospective study (adjusted HR 2.54, 95% CI: 1.32–4.89) [49] as well as in a large study including 1084 patients with colorectal cancer (adjusted HR 1.34, 95% CI: 1.09–1.64) [39]. Given the widespread testing for *KRAS* mutations in patients with colorectal cancer, this genetic information may serve as a readily available marker for VTE risk.

### Nonsmall cell lung cancer

In nonsmall cell lung cancer (NSCLC), various genetic aberrations have been studied for their association with VTE, including ALK fusions, KRAS mutations, and EGFR mutations. The prevalence of these mutations ranges from 4–5% for ALK fusions, 32–38% for EGFR mutations, and up to 30% for KRAS mutations [50–53]. A systematic review of 22 studies with 10 564 NSCLC patients demonstrated that ALK was associated with a twofold increased risk of VTE (RR 1.97, 95% CI: 1.54–2.52), whereas EGFR (RR 0.97, 95% CI: 0.69–1.34, *P* = 0.83) or KRAS mutations (RR 1.27, 95% CI: 0.80–2.02, *P* = 0.32) were not associated with VTE [54,55].

### Other mutations in solid tumors

A large retrospective cohort study including 11 695 patients with cancer evaluated the association of 341 driver mutations in relation with VTE risk [39]. Several driver mutations appeared to be significantly associated with VTE, even when adjusted for age, chemotherapy, history of VTE, metastatic cancer, and anticoagulant use, including STK11 (HR 2.12, 95% CI: 1.55–2.89), CDKN2B (HR 1.45, 95% CI: 1.13–1.85), KEAP1 (HR 1.84, 95% CI: 1.21–2.79), CTNNB1 (HR 1.73, 95% CI: 1.15–2.60), and MET (HR 1.83, 95% CI: 1.15–2.92). Additional research is needed to validate the association of these genes with VTE [39]. In addition, it remains unclear whether these driver mutations are causally associated with VTE, or whether they should be considered an epiphenomenon as markers of an aggressive tumor. This distinction is not relevant for predictive purposes, but studies evaluating a causal link can deepen our understanding of the pathophysiology of cancer-associated thrombosis.

### Circulating tumor DNA

Liquid biopsies to detect circulating tumor DNA (ctDNA) are increasingly used for monitoring disease and guiding treatment. This genetic information may also be used in parallel to improve prediction of cancer-associated VTE. Leveraging circulating DNA from 6030 patients with various cancer types, Jee *et al.* found several genes to be significantly associated with an increased VTE risk independent of cancer type and cancer type specific mutations, including KRAS (adjusted HR 1.65), EGFR (adjusted HR 1.62), CDKN2A (adjusted HR 1.84), KEAP1 (adjusted HR 2.50), STK11 (adjusted HR 1.61), CTNNB1 (adjusted HR 2.70), TP53 (adjusted HR 1.85), and ERBB2 (adjusted HR1.72) [56^▪▪^]. Moreover, detectable ctDNA was an independent VTE predictor compared to undetectable ctDNA (adjusted HR 1.66, 95% CI: 1.30–2.11). A prediction model based on detectable ctDNA, somatic mutations, cell-free DNA concentration, cancer type, and chemotherapy demonstrated better discriminatory ability than the Khorana score (*c*-statistic 0.73 vs. 0.61), suggesting that liquid biopsies have the potential to identify patients at high risk of VTE [56^▪▪^].

## DISCUSSION AND FUTURE PERSPECTIVES

In this review, we have summarized literature on germline and somatic mutations associated with an increased VTE risk in patients with cancer. Genetic profiling of tumor material is becoming widely used for therapeutic and prognostic purposes in patients with cancer [57]. The information obtained on germline and somatic mutations holds promise to ameliorate prediction of VTE in this population. Improvements in DNA sequencing techniques have made it possible to detect circulating tumor DNA at high sensitivity, referred to as liquid biopsies. The decreasing costs of these methods make them more accessible to a larger group of patients [58]. As a result, we can expect a significant increase in available genetic data, which could further enhance the prediction of cancer-associated thrombosis.

Among germline mutations, factor V Leiden and prothrombin G20210A have the strongest and most consistent association with VTE. They increase VTE risk by two-fold in patients with cancer, who already face up to a nine-fold increased risk of VTE. Therefore, the current ASH guidelines suggest to give thromboprophylaxis to cancer patients with known thrombophilia [12]. Targeted testing is only suggested in patients with a family history of VTE. The relatively low prevalence of these mutations also limits the yield of routine testing in clinical practice. Polygenic risk scores, which can incorporate single mutations, may be used to identify a larger proportion of patients as being high risk. The increasing use of tumor sequencing in clinical practice allows the use of germline genetic information without the need for separate testing. Polygenic risk scores have been shown to modestly improve prediction on top of clinical markers, of which the ONCOTHROMB score is the most recent example. Developing a model that incorporates all potential VTE-related SNPs alongside known clinical predictors could further assess the predictive value of germline mutations in cancer patients.

The association of somatic mutations has been studied across different cancer types: IDH1 mutations appear to strongly decrease VTE risk in gliomas, whereas ALK fusions in NSCLC and KRAS mutations in colorectal cancer appear to increase VTE risk. Most somatic mutations are likely to be tumor-specific VTE predictors only, although some are associated with increased VTE risk across multiple cancers. Incorporating these mutations into existing clinical prediction models or developing tumor-specific models could improve the identification of patients at high risk of VTE. Large studies with sufficient statistical power will be needed to develop such models because of the large number of somatic mutations in many types of cancer. A simpler approach is to use the presence of circulating tumor DNA as a marker of VTE risk.

In conclusion, both germline and somatic mutations are independent predictors of VTE in patients with cancer. The growing availability of genetic profiles will provide opportunities for further development and use of improved prediction models in clinical practice.

## Acknowledgements

*None.*

### Financial support and sponsorship

*None.*

### Conflicts of interest

*Competing interest statement: H.W. received funding from MSD, Nordic and Servier and received advisory board honoraria from MSD, Astra Zeneca and Servier, which were transferred to her institute. PK received research funding from Daiichi Sankyo and Roche diagnostics. N.v.E. received advisory board honoraria from Daiichi Sankyo, LEO Pharma, and Bayer, which were transferred to his institute. The other authors have nothing to declare.*

## Supplementary Material

Supplemental Digital Content

## REFERENCES AND RECOMMENDED READING

Papers of particular interest, published within the annual period of review, have been highlighted as:

▪ of special interest
▪▪ of outstanding interest
