# Supplementary material for: The role of germline and somatic mutations in predicting cancer-associated thrombosis: a narrative review
Source: Curr Opin Hematol. 2025 Jan 28;32(3):138–45. doi: 10.1097/MOH.0000000000000861 (PMC11957438; doi:10.1097/MOH.0000000000000861)
Supplement: Supplemental Digital Content [file cohem-32-138-s001.docx]

**Supplementary Table 1.** Search strategy

| Cancer (#1) | "Neoplasms" [Mesh] OR “cancer patient*” [tiab] OR cancer* [tiab] OR neoplas* [tiab] OR malign* [tiab] OR tumor* [tiab]  (N= 5216178) |
| --- | --- |
| Genetics (#2) | "Genetics" [Mesh] OR "Genetic Markers" [Mesh] OR "Genetic Predisposition to Disease" [Mesh] OR "Polymorphism, Single Nucleotide" [Mesh] OR genetic* [tiab] OR “DNA” [tiab]  (N= 2676432) |
| Venous thrombosis (#3) | "Venous Thrombosis"[Mesh] OR "Venous Thromboembolism" [Mesh] OR "Thrombosis" [Mesh] OR "Pulmonary Embolism" [Mesh] OR “cancer associated thrombosis” [tiab] OR “venous thromboembolism” [tiab] OR “venous thrombosis” [tiab] OR “deep vein thrombosis” [tiab] OR “pulmonary embolism” [tiab]  (N= 235941) |
| Search | #1 AND #2 AND #3  (N= 912) |

Screening of the identified articles was performed by one of the authors (MO) using Rayyan (Rayyan, Cambridge, United States) based on titles and abstracts. Studies were included if they: (1) were cohort or case-control studies, (2) included adults, (3) reported on the association between VTE and a genetic predictor in patients with solid cancer, and (4) were published in English. Reference lists of eligible articles were also manually searched.
